# Dataset on critical angular clamping speeds of fasteners

**DOI:** 10.1016/j.dib.2023.110022

**Published:** 2023-12-29

**Authors:** Jeferson Ferreira, Adalto Farias, Fabrizio Leonardi, Ed C. Bordinassi, Roberto Bortolussi, Sergio Delijaicov

**Affiliations:** a University Center FEI– Ignatian Educational Foundation “Padre Saboia de Medeiros”, Av. Humberto de Alencar Castelo Branco, 3972-B, Assunção, São Bernardo do Campo, São Paulo, Brazil; b University Center of Mauá Institute of Technology, Praça Mauá 1, São Caetano do Sul, São Paulo, Brazil; c CEETEPS–State Center of Technological Education “Paula Souza”, Pça. Coronel Fernando Prestes, 30, Bom Retiro, São Paulo, São Paulo, Brazil

**Keywords:** Stick-slip, Preload, Bolted joint, Friction, DOE

## Abstract

This article reports on an experiment that studied the critical angular clamping speeds for fasteners using the Design of Experiments (DOE) methodology and Analysis of Variance (ANOVA). The study aimed to investigate the stick-slip phenomenon, a critical factor limiting the angular speed. The stick-slip was measured using the stick-slip factor, which is defined as the ratio of stick-slip chattering amplitude to frequency. The investigation focused on the factors that affect the stick-slip factor, torque, and clamping force (preload): friction coefficient, clamping angular velocity, cathodic electrodeposition, and hardness of the bolthead bearing plate. Automated predictive algorithms can utilize the data collected from this study to prevent the occurrence of the stick-slip phenomenon in screw clamping processes.

Specifications Table

**Table:** 

Subject	Engineering
Specific subject area	Mechanical engineering and surface engineering concerning coatings and films
Data format	Raw data in .csv file (dataset with numbers) Analyzed data in .csv files and tables with the article
Type of data	Table Image Graph Figure
Data collection	A complete factorial design with four independent factors: friction coefficient (*µ*), angular velocity (*V*), nuts with KTL, and bearing-plate hardness (*h*), in 2 levels and 3 replicates, a total of 48 experimental runs. The dependent variables measured: stick-slip factor (*f_ss_*), preload (P), and torque (T). The screws used follow IS0 898-1 standard, class 10.9, size M10 × 1.50 and 65mm length, with zinc-flake treatment in two friction coefficients. Nuts M10 × 1.50, IS0 898-2 standard, with KTL paint and without.
Data source location	FEI University Center – Ignatian Educational Foundation “Padre Saboia de Medeiros”; São Bernardo do Campo; São Paulo; Brazil
Data accessibility	With the article, and The data is publicly available online at: Repository name: Mendeley Data identification number: 10.17632/jffwrb4pcd.4 Direct URL to data: https://data.mendeley.com/datasets/jffwrb4pcd/4

## 1 Value of the Data

• It is crucial to ensure the reliability and robustness of component unions, especially for parts that are disassembled over time or used in late-stage industrial applications, such as bolted joints.
• Screw-related failures, including issues like fastener breakage or loosening, account for over 80% of recalls in the automotive industry [1].
• The dataset provides valuable information for researchers developing experiments related to assembly parts in industrial applications, such as automotive energy generation, where loosened bolted joints can lead to failures. The data can help identify candidate parameter conditions for these experiments.
• By analyzing the raw data provided, researchers can gain deeper insights into the dynamic relationships between force and torque during clamping. This understanding is critical for overcoming the limitations imposed by the stick-slip phenomenon.
• The dataset also enables the development of automated predictive algorithms by researchers seeking to prevent the occurrence of the stick-slip phenomenon in screw clamping processes. This application enhances the efficiency and reliability of the automated screw-clamping system.

## 2 Background

This study presents a statistical approach to measure the critical velocity at which stick-slip events occur during fastener tightening. The method considers several parameters, such as the coefficient of friction, tightening speed, the presence of KTL (Kathodische TauchLackierung) coating on the nut, and the hardness of the screw-head seating plate material.

**Fig. 1:**
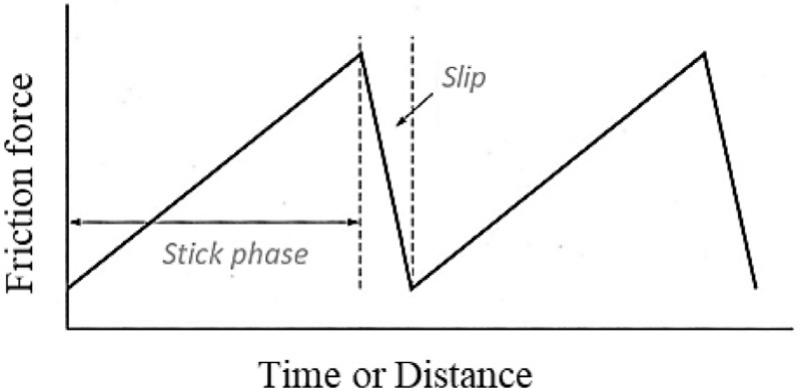
Diagram of stick-slip behavior with a friction force in the function of time or distance. Adapted from [2].

**Fig. 2:**
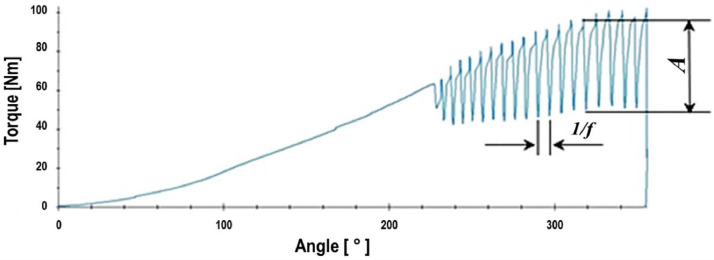
Tightening curve example with characteristics of *stick–slip*.

Fig. 1 illustrates the stick-slip behavior, while Fig. 2 showcases a typical fastener tightening curve with stick-slip. In the plot, A denotes the amplitude, and f represents the frequency of the friction torque series data. Stick-slip is observed both at microscopic and macroscopic scales, and it can propagate from small areas to involve whole surfaces [4]. Researchers such as Bustos and Furlong [5] attribute it to adhesion hysteresis, while Lee et al. [6] relate it to contact stiffness. Other explanations for stick-slip include the elastic deformation of the materials involved and plowing friction [[3], [7]].

Electronic machine-based tightening control relies on torque. Stick-slip can disrupt this, leading to premature screw joint failure [8]. The KTL process is a cathodic coating used in the automotive industry but can alter friction coefficients [9].

The parameters *A* (amplitude) and *f* (friction frequency) were numerically obtained from the raw data for each test and then applied to Eq. 1 to obtain the *f_ss_*.

## 3 Data Description

The datasets related to this article can be accessed by the public in the repository specified in the “Data Accessibility” section found within the Specifications Table. These datasets include the raw data for the torque and force curves obtained during experimentation, plots of such raw data for torque and force, and supplementary tables that support the experimental methodology.

The repository is organized into four folders for data related to the stick-slip phenomenon experiments conducted in Section 3:

The data related to the stick-slip phenomenon experiments conducted in Section 3 are organized into four folders in the repository.

• The “1-raw_data_torque” folder contains raw torque versus angular rotation data collected from 48 tests, the torque behavior during the screw clamping process.
• The “2-raw_data_force” folder contains the raw force versus angular rotation data from the tests and the force dynamics related to torque variations.
• The third folder, labeled “3-raw_data_plots”, contains plots generated from the raw data of the first two folders, providing a visual representation of data trends. Figs. 4 and 5, presented in Section 3 of the data article, illustrate the plotting of the raw data from tests #2 and #7, respectively.
• The “4-DOE_tables” folder contains tables from the Design of Experiments (DOE) conducted in the methodology, summarizing the experimental conditions and results for support, reference, and review.

The raw data files are in CSV format, comma-separated values. The CSV files related to torque contain two data columns: one column has values for angular rotation labeled as “Angle (summary)” in degrees, while the other column is labeled “Torque” with values given in Newton-meters (Nm). Similarly, the CSV files related to force also have two data columns: one with angular rotation values labeled as “Angle (summary)” in degrees and another labeled “Force” with values given in kilonewtons (kN).

The stick-slip factor measures how often stick-slip occurs, which is the ratio of the chattering amplitude to frequency. Eq. (1) defines the stick-slip factor. Angular velocity is important in industrial processes, and staying above a critical value can prevent stick-slip [10]. Stick-slip has been studied in various contexts [11], [12], [13], [14]. This study used a DOE (Design of Experiments) to determine the correlation between critical stick-slip factors and angular velocities. Independent variables like friction coefficient, angular velocity, surface treatment, and material hardness were varied to assess their influence on stick slip.

(1) $$f_{ss}=\frac{A}{f}\left\{ \begin{array}{c} if\;A=0\;then\;f_{ss}=0\;and\;there\;is\;no\;stick-slip\;\\ if\;A>0\;and\;f\neq 0\left\{ \begin{array}{c} if\;f_{ss}\geq f_{ss,crit}\;then\;there\;is\;no\;stick-slip\\ if\;f_{ss}<f_{ss,crit}\;then\;there\;is\;stick-slip \end{array}\right. \end{array}\right.$$

The study utilized a complete factorial experimental design with four independent variables, as mentioned in Table 1. These variables were the coefficient of friction, angular velocity, nuts with KTL, and bearing-plate hardness, each with two levels. The study was conducted with three replicates, resulting in 48 experiments run (16 in each replicate), as specified in Table 2.

**Table 1:** The DOE's independent variables and levels.

Independent Variables	Factors			
	*µ*	*V*	KTL nut	*h*
		rad/min		HV
Levels	0.10	10	-1	135
	0.20	200	1	430

**Table 2:** Data obtained through planning the DOE experiment.

*Run #*	*Randomized*	*Replicate*	*µ*	*V*	*KTL nut*	*h*	*f_ss_*	*P*	*T*
				[rad/min]	paint	[HV]		[kN]	[Nm]
1	7	1	0.1	10	−1	135	0	49.1	83.6
2	22	2	0.2	10	−1	135	0	43.8	119.7
3	6	3	0.1	200	−1	135	0	54.7	73.8
4	48	1	0.2	200	−1	135	0	48.5	101.2
5	11	2	0.1	10	1	135	0	48.4	81.8
6	12	3	0.2	10	1	135	0	40.2	114.3
7	9	1	0.1	200	1	135	0.74	51.4	66.0
8	19	2	0.2	200	1	135	1.83	45.7	86.0
9	29	3	0.1	10	−1	430	0	54.8	76.9
10	18	1	0.2	10	−1	430	0	44.1	114.2
11	23	2	0.1	200	−1	430	0	56.1	61.1
12	20	3	0.2	200	−1	430	0	47.5	96.7
13	41	1	0.1	10	1	430	0	54.2	76.7
14	28	2	0.2	10	1	430	0	42.5	117.9
15	21	3	0.1	200	1	430	0.8	50.6	59.0
16	38	1	0.2	200	1	430	1.6	47.7	74.0
17	3	2	0.1	10	−1	135	0	48.7	84.2
18	42	3	0.2	10	−1	135	0	45.0	122.4
19	34	1	0.1	200	−1	135	0	53.4	69.4
20	17	2	0.2	200	−1	135	0	47.6	96.3
21	43	3	0.1	10	1	135	0	49.2	82.7
22	31	1	0.2	10	1	135	0	43.5	119.3
23	25	2	0.1	200	1	135	0.55	52.1	64.0
24	35	3	0.2	200	1	135	1.83	43.9	82.0
25	37	1	0.1	10	−1	430	0	53.9	77.6
26	10	2	0.2	10	−1	430	0	43.8	113.8
27	24	3	0.1	200	−1	430	0	56.6	62.5
28	39	1	0.2	200	−1	430	0	48.6	101.0
29	36	2	0.1	10	1	430	0	54.8	71.7
30	40	3	0.2	10	1	430	0	42.2	117.4
31	47	1	0.1	200	1	430	0.91	51.1	62.0
32	1	2	0.2	200	1	430	1.8	45.4	70.0
33	4	3	0.1	10	−1	135	0	50.5	82.2
34	27	1	0.2	10	−1	135	0	44.3	113.6
35	16	2	0.1	200	−1	135	0	54.3	67.5
36	13	3	0.2	200	−1	135	0	46.8	95.9
37	46	1	0.1	10	1	135	0	49.8	80.2
38	30	2	0.2	10	1	135	0	43.6	116.7
39	8	3	0.1	200	1	135	0.44	49.7	61.0
40	14	1	0.2	200	1	135	2.09	44.8	75.0
41	45	2	0.1	10	−1	430	0	53.4	76.7
42	15	3	0.2	10	−1	430	0	45.0	118.7
43	44	1	0.1	200	−1	430	0	56.8	64.0
44	26	2	0.2	200	−1	430	0	46.8	94.5
45	5	3	0.1	10	1	430	0	52.9	74.4
46	2	1	0.2	10	1	430	0	44.5	120.6
47	33	2	0.1	200	1	430	0.67	58.7	54.0
48	32	3	0.2	200	1	430	2.44	45.4	73.0

**Fig. 3:**
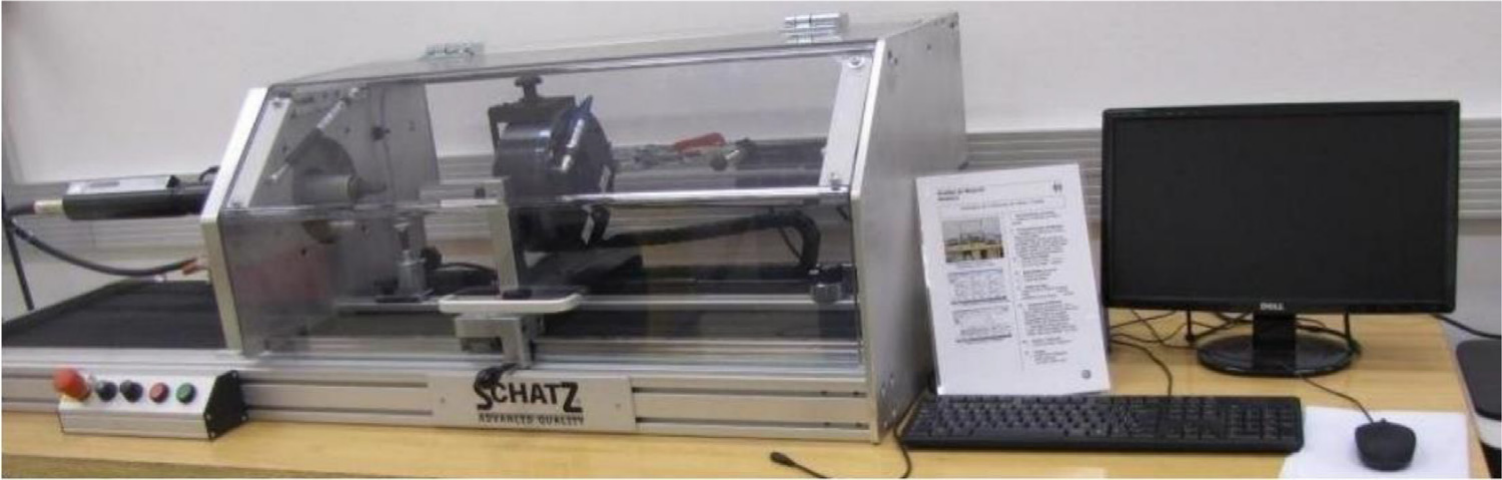
The friction, torque, and force coefficient analyzer equipment.

The equipment shown in Fig. 3 is a screwdriver with a maximum torque sensor of 160 Nm and a maximum angular velocity (V) of 340 rpm. To plan the experiment, we designated a low-level specification of 10 rpm and a high-level specification of 200 rpm to avoid the screwdriver's lower and upper limits. We used M10 × 1.50 screws with zinc-flake surface treatment in two conditions of friction coefficients (µ): one with a low-level (µ = 0.10) and the other with a high-level (µ = 0.20) to represent the limits of the zinc-flake surface treatment. We used M10 × 1.50 nuts with KTL paint (high level: 1) and without KTL paint (low level: -1). We chose steel bearing plates with hardness (h) of 135 HV for the low level and 430 HV for the high level to set the screw head.

The 48 experiments were conducted using a randomized sampling technique to ensure that all factors not accounted for in the design were evenly distributed. In Table 2, the column labeled “Randomized” shows the original randomized order for the setup design.

The dependent variables measured were the stick-slip factor (*f_ss_*), preload (*P*), and torque (*T*).

The numerical data obtained through the DOE are presented in Table 2; the torque (T), force (P), and *f_ss_* values were derived from the torque and force curves obtained during experimental trials, all of which are available in the designated repository.

The DOE method allows us to statistically analyze the influence of each variable and their interactions in stick-slip. To conduct this analysis, we used the Statistica® software Version 8, employed correlation diagrams, and used the analysis of variance tool (ANOVA).

An ANOVA evaluation was conducted to support the raw data available at the repository. The information in Table 3, Table 4, Table 5, Table 6 can be used by a researcher who intends to apply another approach to this data for comparison purposes.

**Table 3:** ANOVA for stick-slip factor *f_ss_.*

Source	DF	Seq SS	Contribution	Adj SS	Adj MS	*F*-value	*p*-value
Model	15	19.3182	94.06%	19.3182	1.28788	33.77	0
Linear	4	10.949	53.31%	10.949	2.73726	71.78	0
*µ*	1	1.6688	8.13%	1.6688	1.6688	43.76	0
*V*	1	5.0505	24.59%	5.0505	5.05052	132.44	0
*KTL*	1	4.2186	20.54%	4.2186	4.2186	110.62	0
*h*	1	0.0111	0.05%	0.0111	0.0111	0.29	0.593
2-Way Interactions	6	7.1166	34.65%	7.1166	1.1861	31.1	0
*µ x V*	1	1.6688	8.13%	1.6688	1.6688	43.76	0
*µ x KTL*	1	1.2065	5.87%	1.2065	1.2065	31.64	0
*µ x h*	1	0.0039	0.02%	0.0039	0.00385	0.1	0.753
*V x KTL*	1	4.2186	20.54%	4.2186	4.2186	110.62	0
*V x h*	1	0.0111	0.05%	0.0111	0.0111	0.29	0.593
*KTL x h*	1	0.0078	0.04%	0.0078	0.00775	0.2	0.655
3-Way Interactions	4	1.2354	6.01%	1.2354	0.30884	8.1	0
*µ x V x KTL*	1	1.2065	5.87%	1.2065	1.2065	31.64	0
*µ x V x h*	1	0.0039	0.02%	0.0039	0.00385	0.1	0.753
*µ x KTL x h*	1	0.0173	0.08%	0.0173	0.01725	0.45	0.506
*V x KTL x h*	1	0.0078	0.04%	0.0078	0.00775	0.2	0.655
4-Way Interactions	1	0.0173	0.08%	0.0173	0.01725	0.45	0.506
*µ x V x KTL x h*	1	0.0173	0.08%	0.0173	0.01725	0.45	0.506
Error	32	1.2203	5.94%	1.2203	0.03814		
Total	47	20.5386	100.00%

**Table 4:** ANOVA for preload P.

Source	DF	Seq SS	Contribution	Adj SS	Adj MS	*F*-value	*p*-value
Model	15	924.3	92.83%	924.3	61.62	27.61	0
Linear	4	870.621	87.44%	870.621	217.655	97.51	0
*µ*	1	705.333	70.84%	705.333	705.333	316	0
*V*	1	80.083	8.04%	80.083	80.083	35.88	0
*KTL*	1	36.401	3.66%	36.401	36.401	16.31	0
*h*	1	48.803	4.90%	48.803	48.803	21.86	0
2-Way Interactions	6	7.1166	34.65%	7.1166	1.1861	31.1	0
*µ x V*	1	2.253	0.23%	2.253	2.253	1.01	0.323
*µ x KTL*	1	0.188	0.02%	0.188	0.188	0.08	0.774
*µ x h*	1	28.213	2.83%	28.213	28.213	12.64	0.001
*V x KTL*	1	8.841	0.89%	8.841	8.841	3.96	0.055
*V x h*	1	2.803	0.28%	2.803	2.803	1.26	0.271
*KTL x h*	1	1.021	0.10%	1.021	1.021	0.46	0.504
3-Way Interactions	4	10.219	1.03%	10.219	2.555	1.14	0.353
*µ x V x KTL*	1	3.968	0.40%	3.968	3.968	1.78	0.192
*µ x V x h*	1	5.603	0.56%	5.603	5.603	2.51	0.123
*µ x KTL x h*	1	0.608	0.06%	0.608	0.608	0.27	0.605
*V x KTL x h*	1	0.041	0.00%	0.041	0.041	0.02	0.893
4-Way Interactions	1	0.141	0.01%	0.141	0.141	0.06	0.803
*µ x V x KTL x h*	1	0.141	0.01%	0.141	0.141	0.06	0.803
Error	32	71.427	7.17%	71.427	2.232		
Total	47	995.727	100.00%

**Table 5:** ANOVA for Torque T.

Source	DF	Seq SS	Contribution	Adj SS	Adj MS	*F*-value	*p*-value
Model	15	20207.3	98.64%	20207.3	1347.2	155.08	0
Linear	4	18567.7	90.64%	18567.7	4641.9	534.35	0
*µ*	1	11463.9	55.96%	11463.9	11463.9	1319.65	0
*V*	1	6256.3	30.54%	6256.3	6256.3	720.19	0
*KTL*	1	590.8	2.88%	590.8	590.8	68.01	0
*h*	1	256.7	1.25%	256.7	256.7	29.55	0
2-Way Interactions	6	1301.4	6.35%	1301.4	216.9	24.97	0
*µ x V*	1	660.1	3.22%	660.1	660.1	75.98	0
*µ x KTL*	1	117.8	0.58%	117.8	117.8	13.56	0.001
*µ x h*	1	51.7	0.25%	51.7	51.7	5.95	0.02
*V x KTL*	1	460	2.25%	460	460	52.96	0
*V x h*	1	10.8	0.05%	10.8	10.8	1.25	0.273
*KTL x h*	1	1	0.00%	1	1	0.11	0.741
3-Way Interactions	4	288.2	1.41%	288.2	72	8.29	0
*µ x V x KTL*	1	253	1.24%	253	253	29.12	0
*µ x V x h*	1	12.8	0.06%	12.8	12.8	1.47	0.233
*µ x KTL x h*	1	3.9	0.02%	3.9	3.9	0.44	0.51
*V x KTL x h*	1	18.5	0.09%	18.5	18.5	2.13	0.154
4-Way Interactions	1	50	0.24	50	50	5.76	0.022
*µ x V x KTL x h*	1	50	0.24	50	50	5.76	0.022
Error	32	278	1.36%	278	8.7		
Total	47	20485.3	100.00%

**Table 6:** Data of the ANOVA for *stick–slip* response *f_ss_*.

	Regression Coeff.	Std. Error	t(20)	p-value	-95% Confid. Limit	+95% Confid. Limit
Constant	0.029561	0.163395	0.18092	0.858251	-0.31127	0.370397
*µ*	−0.656140	1.033399	−0.63493	0.532669	−2.81177	1.499493
*V*	−0.002956	0.001154	−2.56179	0.018599	−0.00536	−0.000549
*µ* and *V*	0.065614	0.007298	8.99054	0.000000	0.05039	0.080838

Table 3 can be used to observe the occurrence of the stick-slip effect through the mechanism of rough surfaces. It is known that surface variations or imperfections can influence the stick-slip effect's appearance [4].

Table 4 can be used to observe the influential variables tested in the preload response P.

Table 5 can be used to confirm the influential variables tested in the torque response T.

The statistical data supported the decision to remove the independent variable hardness of the support plate and nut in the stick-slip model. Subsequently, new analyses were conducted using the Statistica® software, and ANOVA was employed to derive the response surface equation for the stick-slip factor. This equation is presented in Table 6 and can be denoted as Eq. 4.

### 3.1 Experimental design, materials and methods

The objective of the methodology was to determine the minimum angular velocity, known as the critical velocity, required in bolted joints to prevent stick slip and enable the tightening of threaded fasteners to achieve a preload within the expected range. The statistical approach used success cases without stick-slip and failure cases with stick-slip, quantified by the stick-slip factor (*f_ss_*), which caused the interruption of the tightening process. In summary, the method can be described as follows:

(1) Choose the independent variables (*µ, V, KTL, h*) and their levels regarding the DOE;
(2) Adopt the stick-slip factor (*f_ss_*) as the dependent variable;
(3) Include both the clamping preload force (*P*) and torque (*T*) as dependent variables;
(4) Perform the DOE;
(5) Analyze the *f_ss_* ANOVA identifying the significant independent variables;
(6) Keep only the significant independent variables and perform a new regression;
(7) Plot the contour curves of the analytic *f_ss_* surface response;
(8) Perform a new experiment to identify the contour line, which is the boundary for stick-slip occurrences;
(9) Derive a function of the critical velocity *V_crit_* as a function of the friction coefficient;
(10) Use the ANOVA of *P* and *T* to predict the preload and final torque values after the complimentary tests.

The screws utilized adhere to the IS0 898-1 standard, which denotes a resistance class of 10.9, with specifications of M10 × 1.50 × 65, featuring a zinc-flake surface treatment. These screws exhibit two friction coefficients: one labeled as low-level (µ = 0.10) and the other as high-level (µ = 0.20). The high level represents a coating condition comprising one layer of basecoat and one layer of topcoat. In contrast, the low-level represents the same coating condition with adding a lubricant layer. A representative batch sample was evaluated to ensure quality, with the friction coefficient controlled according to standardized testing and calculation procedures. Upon metallographic analysis, the screw structure was found to be tempered martensite, with a measured hardness in the range of 342-347 HV.

In compliance with the IS0 898-2 minimum load resistance standard, we utilized Nuts M10 × 1.50 with KTL paint (high level: 1) and without (low level: -1). Our selection of steel-bearing plates to secure the screw head was based on two hardness levels: low (135 HV) and high (430 HV).

To measure the preload and torque, we utilized a Bosch screwdriver device (Fig. 3) and followed the procedure outlined in the industry-standard VDI-2230 (2003) to obtain the friction coefficient of the screws. For accuracy, all tests were conducted in a controlled, air-conditioned laboratory with equipment calibrated to the rigorous standards and methodologies of the Brazilian Calibration Network (RBC).

**Fig. 4:**
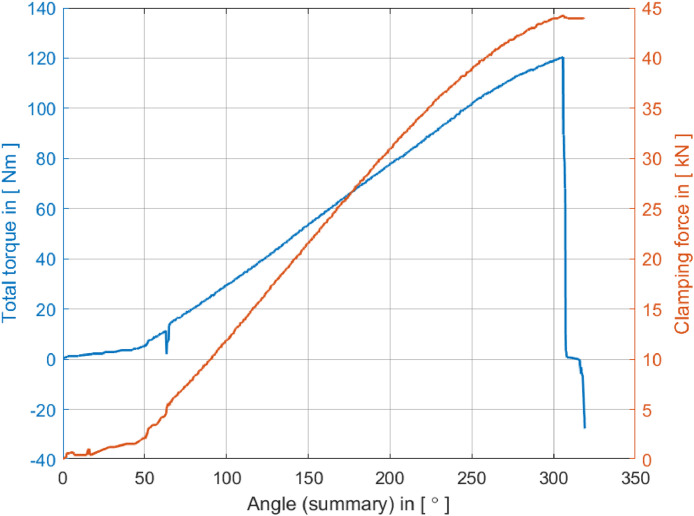
Tightening curve acquisition for test #2 with no stick–slip.

**Fig. 5:**
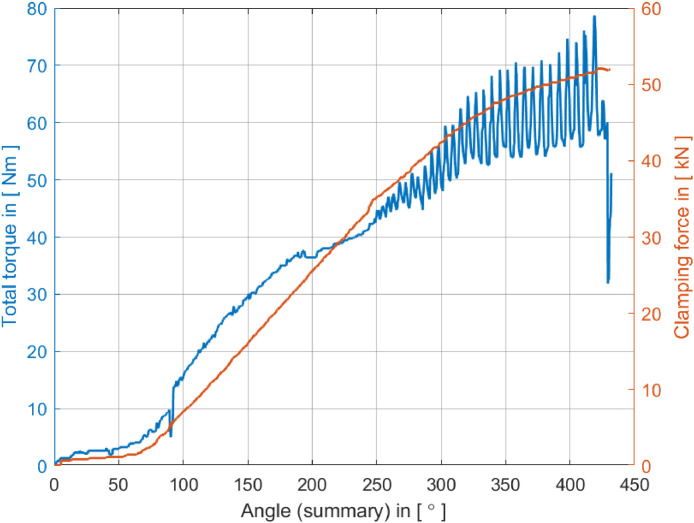
Tightening curve acquisition for test #7 with *stick–slip* characteristics.

We utilized the torque and angle techniques for this experiment, and the analysis equipment was calibrated periodically to ensure accurate results. Each test involved a constant tightening torque of 40 Nm and an angular increment of 180°, with the bearing plate being replaced for each test with one of the same characteristics. The software used for acquiring torque, angle, and clamping force was testXpert II, developed by Schatz® Advanced Quality for this application. This software allowed us to conFig. all test steps, including the geometric characteristics of the screw and thread. At the end of tests 2 and 7, we generated graphical plots, as shown in Fig. 4, Fig. 5, respectively.

Eqs. (2) to (5) present mathematical relationships using only significant factors from the DOE methodology.

Eq. (2) demonstrates the regression model for the Stick-Slip Factor, which is derived from Table 3. This model presented R^2^ = 97.21%, R^2^_adj_ = 96.72%, and R^2^_pred_ = 95.98%. The regression equation of the stick-slip factor makes it possible to draw the response surface for the phenomenon.

(2) $$\begin{array}{ccc} f_{ss} & = & 0.015-0.328\cdot \mu -0.0015\cdot V+0.0148\cdot \text{KTL}+0.033\cdot \mu \cdot V\\ & & -\;0.328\cdot \mu \cdot \text{KTL}-0.0015\cdot V\cdot \text{KTL}+0.033\cdot \mu \cdot V\cdot \text{KTL} \end{array}$$

Eq. (3) presents the resulting regression model for Preload from Table 4. This model presented R^2^ = 91.16%, R^2^_adj_ = 89.86% and R^2^_pred_ = 87.88%.

(3) P=52.62−47.3·μ+0.0136·V−0.3965·KTL+0.0224·h−0.104·μ·h−0.0045·V·KTL

Eq. (4) presents the resulting regression model for Torque from Table 5. This model presented R^2^ = 98.33%, R^2^_adj_ = 97.87% and R^2^_pred_ = 97.11%.

(4) $$\begin{array}{ccc} & & T=51.05+351.8\cdot \mu -0.0025\cdot V-3.0\cdot \text{KTL}-0.0364\cdot h-0.7833\cdot \mu \cdot V\\ & & \;+19.44\cdot \mu \cdot \text{KTL}+0.139\cdot \mu \cdot h\\ & & \;+0.0404\cdot V\cdot \text{KTL}-0.4204\cdot \mu \cdot V\cdot \text{KTL}-0.00023\cdot \mu \cdot V\cdot \text{KTL}\cdot h \end{array}$$

Eq. (5) presents the resulting regression model from Table 6, employing only *µ* and *V*. This model presented R^2^ = 96.28% and R^2^_adj_ = 95.72% and R^2^_pred_ = 96.64%.

(5) $$f_{ss}=0.0296-0.6561\cdot \mu -0.00296\cdot V+0.0656\cdot \mu \cdot V$$

## Limitations

Two critical factors affecting experimental design and data collection limitations are the quality of zinc-flake surface treatment on bolts and the thickness of the KTL layer on nuts, which directly affect friction coefficient (µ), force, and torque.

For Experimental Design:

• The coefficient µ was not effectively evaluated before the execution of tests. Instead, was considered fixed in 0.10 and 0.20 levels.
• The bath tolerances control of the zinc-flake surface treatment process was not automated, allowing variation of up to +/-0.02. In practice, µ could range from 0.08-0.12 for the low level and 0.18-0.22 for the high level.
• No thickness control was performed on the KTL nuts layer due to the difficulty in measuring the reduced dimensions of an M10 nut layer. Variations in the thickness during processing could also occur.

For Data Collection:

• Variations in µ may interfere with the outcome of force and torque curve values, altering the behavior observed, as they are directly associated with this factor.
• The force and torque results might exhibit a slight bias due to µ not being directly evaluated through measurement but instead inferred from a treatment process result.
• Variations in the KTL layer may interfere with the occurrence of the stick-slip phenomenon.

Suggestions for Future Research:

• Applying the zinc-flake surface treatment in an automated line with enhanced controls to reduce µ variation.
• Measure the µ factor in bolts with characterization tests like the pin-on-disc method.
• Perform new tests on larger bolts/nuts (e.g., above M24, prevalent in the wind industry) to measure the KTL layer without causing damage to the nut.”

## Ethics Statement

The authors affirm that they have carefully reviewed and adhere to the ethical guidelines for publication in Data in Brief. Furthermore, they confirm that the present study does not involve human subjects, animal experimentation, or the utilization of data sourced from social media platforms.

## CRediT authorship contribution statement

**Jeferson Ferreira:** Investigation, Formal analysis, Writing – original draft. **Adalto Farias:** Formal analysis, Data curation, Writing – review & editing. **Fabrizio Leonardi:** Data curation, Writing – review & editing. **Ed C. Bordinassi:** Investigation, Writing – review & editing. **Roberto Bortolussi:** Writing – original draft. **Sergio Delijaicov:** Conceptualization, Project administration, Writing – review & editing.

## Data Availability

Dataset on force and torque for fastener clamping (Original data) (Mendeley Data)
